# Development of a literature informed Bayesian machine learning method for feature extraction and classification

**DOI:** 10.1186/1471-2105-16-S15-P9

**Published:** 2015-10-23

**Authors:** Behrouz Madahian, Lih Yuan Deng, Ramin Homayouni

**Affiliations:** 1 Department of Mathematical Sciences, University of Memphis, Memphis, TN 38152, USA; 2 Bioinformatics Program, University of Memphis, Memphis, TN 38152, USA; 3 Department of Biological Sciences, University of Memphis, Memphis, TN 38152, USA

## Background

Gene expression profiling is a powerful approach to identify markers for classification of samples; however, it has major limitations that hinder performance. Typically, a large number of variables are assessed compared to relatively small sample sizes. In addition, it is difficult to identify biologically informative markers which have high predictive power[1-3]. Thus, the goal of this study was to develop a machine learning approach that is able to bridge classification accuracy and biological function.

## Materials and methods

We developed a **L**iterature aided **S**parse **B**ayesian **G**eneralized Linear model which utilizes **G**eneralized Double Pareto (LSBGG) prior to induce shrinkage in terms of the number of covariates. Importantly, instead of using uninformed hyper parameters for the prior distributions, we adjusted the hyper parameters based on the ranking of the genes by GeneIndexer (Quire Inc. Memphis, TN) with respect to ‘cancer’ keyword query. This unique approach controls shrinkage imposed on genes based on biological function extracted from the literature. The model was applied to a leukemia data set from Golub et al.[4]. The dataset was split into training and test groups and classification performance was evaluated on the test group. The top 500 highly differentially expressed genes were used for the modeling step.

## Results

**Figure 1:**
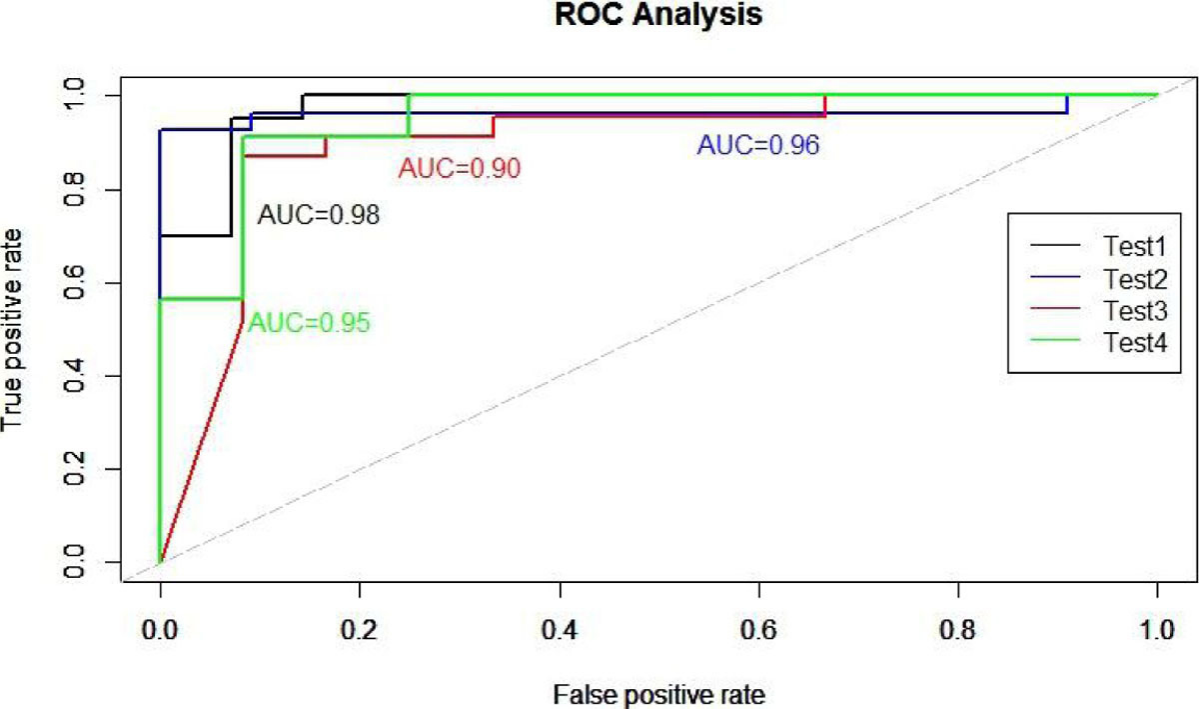
ROC curves for the models with or without literature imposed shrinkage. The area under the curve (AUC) for the model incorporating literature (Tests 1 &2) is higher than the model without incorporating literature (Tests 3 &4).

**Figure 2:**
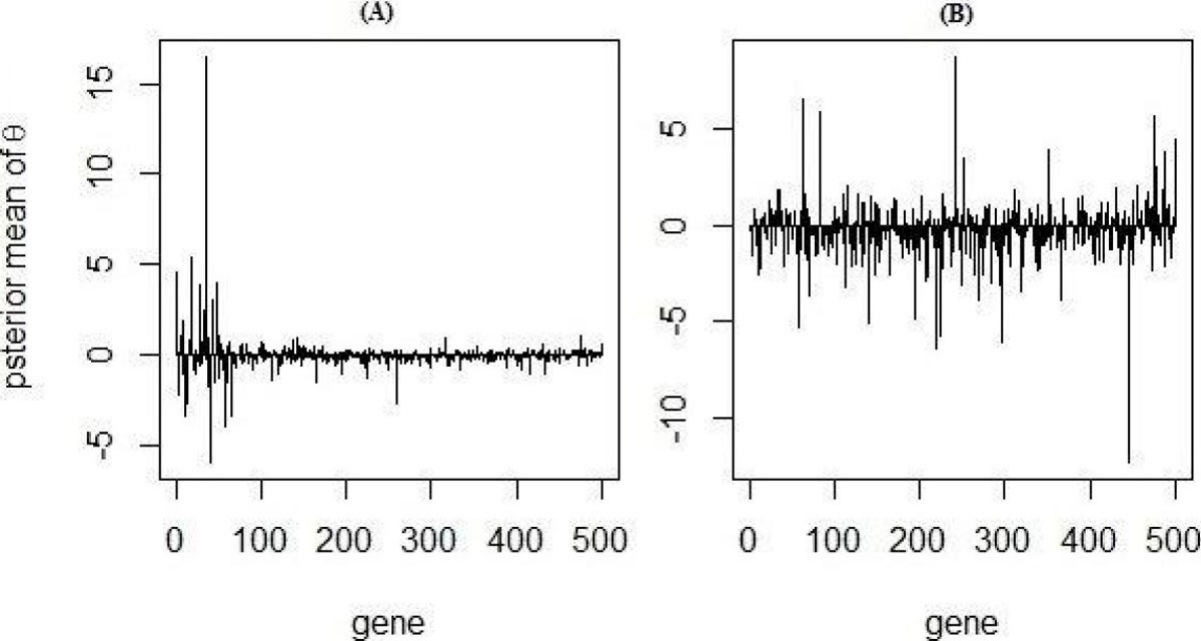
Relationship between the posterior mean of *θs* and biological relevance. The posterior mean of *θ* (Y-axis) is shown for the top 500 genes (X-axis) when using a model with (A) or without (B) incorporation of literature to control shrinkage. There is a clear association between the estimated θ in the model and association with cancer in the literature.

Using the top 10 genes obtained from LSBGG, we were able to achieve 91% classification accuracy in the test group. When the training and test datasets were switched, we obtained 92% classification accuracy. In contrast, the model without biological information achieved 91% and 86% classification accuracies in the two test scenarios (Table 1). Consistent with these results, Receiver Operating Characteristic (ROC) analysis showed better performance when shrinkage was imposed using the literature (Figure 1). Notably, we found that the posterior mean of *θ* was higher for genes which were functionally related to cancer in the biomedical literature (Figure 2).

**Table 1:** Classification accuracy, sensitivity and specificity of the model including (LSBGG) or excluding literature.

	Including Literature		Excluding Literature	
	
	Test1	Test2	Test3	Test4
Measure				
**Accuracy**	91	92	91	86
**Sensitivity**	100	89	100	91
**Specificity**	79	100	75	75

## Conclusions

This demonstrates that while LBSGG performs slightly better in classification of samples, it uses more biologically informative genes, and hence may simultaneously provide insights into the mechanisms underlying the phenotype of interest.
